# A Genome-Wide siRNA Screen to Identify Host Factors Necessary for Growth of the Parasite *Toxoplasma gondii*

**DOI:** 10.1371/journal.pone.0068129

**Published:** 2013-06-28

**Authors:** Lindsey A. Moser, Angela M. Pollard, Laura J. Knoll

**Affiliations:** 1 Department of Medical Microbiology and Immunology, University of Wisconsin-Madison, Madison, Wisconsin, United States of America; 2 Agile Sciences, Inc., Raleigh, North Carolina, United States of America; Centre National de la Recherche Scientifique, France

## Abstract

*Toxoplasma gondii* is an obligate intracellular parasite that is able to infect virtually any nucleated cell of all warm-blooded animals. The host cell factors important for parasite attachment, invasion, and replication are poorly understood. We screened a siRNA library targeting 18,200 individual human genes in order to identify host proteins with a role in *T. gondii* growth. Our screen identified 19 genes whose inhibition by siRNA consistently and significantly lowered parasite replication. The gene ontology categories for those 19 genes represented a wide variety of functions with several genes implicated in regulation of the cell cycle, ion channels and receptors, G-protein coupled receptors, and cytoskeletal structure as well as genes involved in transcription, translation and protein degradation. Further investigation of 5 of the 19 genes demonstrated that the primary reason for the reduction in parasite growth was death of the host cell. Our results suggest that once *T. gondii* has invaded and established an infection, global changes in the host cell may be necessary to reduce parasite replication. While siRNA screens have been used, albeit rarely, in other parasite systems, this is the first report to describe a high-throughput siRNA screen for host proteins that affect *T. gondii* replication.

## Introduction

*Toxoplasma gondii* is an obligate intracellular parasite with a complex life cycle that can include both sexual and asexual stages. While sexual replication occurs only in felines, the asexual cycle can occur in all warm-blooded animals, including humans. The asexual forms of *T. gondii* include tachyzoites, the fast growing form found primarily during acute infection, and bradyzoites, the encysted form responsible for maintaining a chronic infection. After ingestion of bradyzoite cysts, the parasite reverts to its tachyzoite form, which disseminates throughout the body. Tachyzoites actively invade host cells, forming a parasitophorous vacuole where the parasites rapidly replicate until the host cell lyses, releasing more tachyzoites. Newly released tachyzoites repeat the cycle in subsequent cells until immune pressure and other poorly understood physiological cues trigger the parasite to switch to bradyzoites and establish a chronic infection (reviewed in [1]).

Microarray and proteomic studies have been used to examine host cell responses to *T. gondii* infection. Transcriptional profiling of host cells by microarray analysis demonstrated upregulation of host kinases, cell surface antigens, and proteins regulating apoptosis and cell cycle control after infection with *T. gondii* [2], [3]. Microarray analysis has also demonstrated upregulation of immune response-associated genes and global disruptions in interferon gamma and NF-kB signaling in infected cells [2], [3], [4], [5], [6]. Proteomic analysis has revealed alterations of vital pathways, such as mitosis, glycolysis, and apoptosis [7]. Adding to its complexity, the cellular effects of *T. gondii* infection appear to be parasite strain and host cell specific [8]. A global investigation of the host proteins required for *T. gondii* attachment, invasion, and growth will be necessary for full understanding of *T. gondii* interactions with the host cell.

RNA interference (RNAi) has been used extensively to identify and dissect the intimate interactions between host cells and intracellular pathogens. The availability of genome-wide human siRNA libraries allows for high-throughput screening of the entire genome; this technology has been applied successfully to viruses, bacteria and, less frequently, parasites. For example, Karlas *et al*. [9] used a genome-wide siRNA screen to identify 287 host factors that impact influenza A replication. Host cell molecules responsible for *Mycobacterium tuberculosis* intracellular survival and regulation of bacterial load in macrophages were identified using large-scale siRNA screens [10], [11]. A genome-wide siRNA screen identified 162 genes important for growth and persistence of the parasite *Trypanosoma cruzi* [12]. siRNA silencing of the human hepatoma cell kinome during *Plasmodium berghei* sporozoite infection identified five proteins involved in parasite replication [13]. A large-scale RNAi strategy to identify host factors necessary for *T. gondii* growth has not yet been reported.

In this study, we employed an RNAi approach to identify host genes involved in *T. gondii* infection. We screened a human genome siRNA library of approximately 18,200 genes to identify host factors necessary for *T. gondii* infection in HeLa cells. The silencing of 19 human genes resulted in decreased *T. gondii* replication; five were of these were further investigated.

## Materials and Methods

### Cell Culture and Growth of Parasites

*T. gondii* RHΔHXGPRT [14] were maintained as tachyzoites by passage on monolayers of human foreskin fibroblasts (HFFs; ATCC). The HeLa cell line (ATCC) was used for siRNA experiments because immortalized cell lines are more consistent and easily transformed than primary cells such as HFFs. All host cells were grown at 37°C with 5% CO_2_ in complete media composed of Dulbecco’s Modified Eagle Medium (DMEM; Gibco) with 1% penicillin-streptomycin, 2 mM L-glutamine, and 10% fetal bovine serum.

### Construction of *T. gondii* Expressing mCherry

*T. gondii* RHΔHXGPRT (1×10^7^) was electroporated with 25 µg tub-mCherry [15] linearized with KpnI. The population was selected with chloramphenicol to isolate stable transformants. Individual clones were isolated by limiting dilution and screened by fluorescence microscopy for expression of mCherry. Clones were examined by Southern hybridization to verify unique insertion sites (data not shown). Four unique clones were further investigated.

### Growth Assay for *T. gondii*

Insertion of mCherry into the genome could affect the growth rate of the parasite. To isolate a clone with normal growth characteristics, the growth rate of individual RHΔHXGPRT::mCherry clones was compared to that of the parental strain RHΔHXGPRT. HeLa cells grown on coverslips were infected with 1×10^5^ parasites for 12 or 24 hours. The cells were fixed with 3% formaldehyde for 20 minutes, then permeabilized and blocked in PBS containing 3% BSA and 0.2% TritonX-100. The number of parasites per vacuole was noted in 100 vacuoles per coverslip. RHΔHXGPRT::mCherry 9, a clone with a wild type growth rate (data not shown), was selected for use in subsequent experiments.

**Figure 1:**
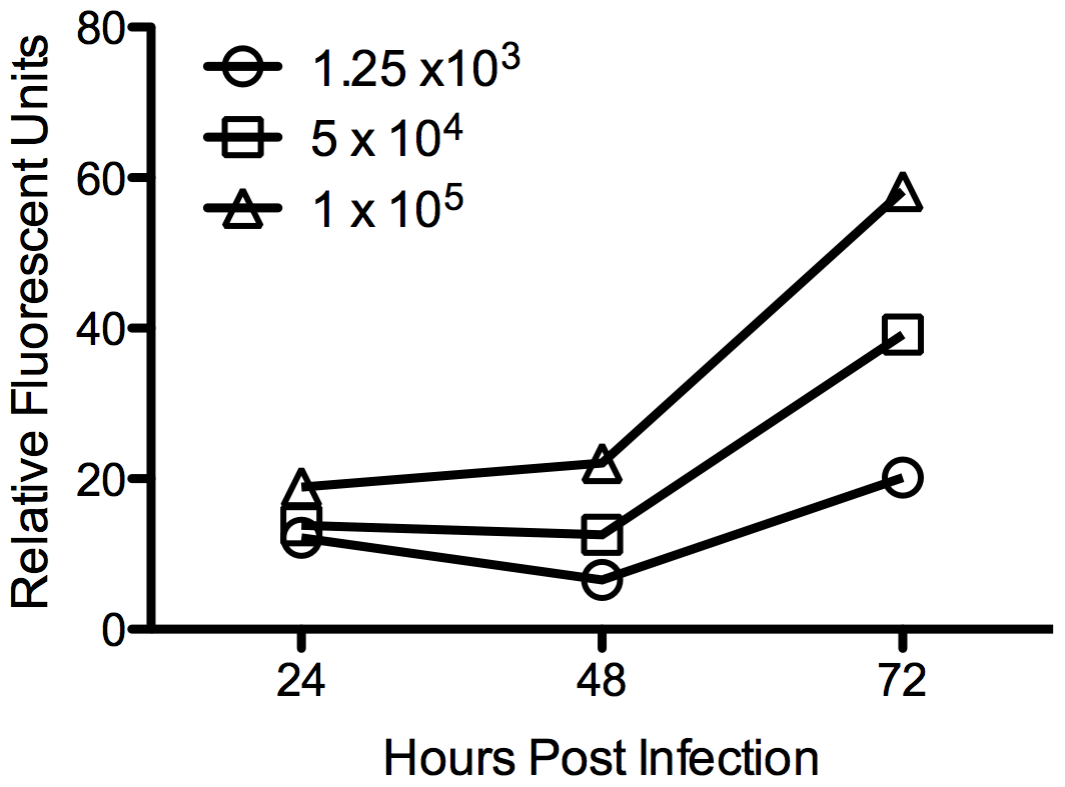
Fluorescence increases over time and with increased *T. gondii* inoculum. HeLa cells were infected with 1.25×10^3^ (circles), 5×10^4^ (squares), or 1×10^5^ (triangles) mCherry-expressing *T. gondii*. Fluorescent measurements were taken at 24, 48, and 72 hours post infection.

To identify the relationship between parasite number and fluorescence, HeLa cells were plated in a 96 well plate (7000/well) and allowed to reach confluency. Confluent monolayers were infected with 1×10^4^/ml–1×10^5^/ml RHΔHXGPRT::mCherry and fluorescence (excitation: 589 nm, emission: 615 nm) was read every 24 hours until 72 hours post infection (hpi) using a SpectraMax Gemini EM fluorometer (Molecular Devices). Fluorescence increased with increasing numbers of parasites and over time until 72 hours (Fig. 1), when the parasites lysed from the monolayer. A higher infectious dose (2.5×10^4^ parasites/well) was used to ensure sufficient sensitivity in the fluorescence signal to detect alterations in parasite growth.

### Optimization of siRNA Procedure

In order to optimize the concentrations of transfection reagent and siRNA, the viability of HeLa cells transfected with varying concentrations of transfection reagent and siRNAs was assessed. HeLa cells were plated at 7000 cells/well in 96 well plate and allowed to obtain ∼75% (∼1.6×10^4^ cells/well) confluency after 24 hours. Transfections were performed using Mirus *Trans*IT-TKO Transfection Reagent according to the manufacturer’s instructions. Briefly, 9 µl of Opti-MEM (Gibco) was incubated with *Trans*IT-TKO Transfection reagent (0.25 µl or 0.35 µl) for 20 minutes at room temperature. Qiagen’s AllStars Hs Cell Death Control or AllStars Negative Control siRNAs were added to a final concentration of 0 nM, 12.5 nM, 25 nM, or 50 nM and incubated at room temperature for 20 minutes. Complete medium (44 µl) was added to the transfection reagent/siRNA mixture and then added to HeLa cells previously seeded in a 96 well plate. Plates were incubated at 37°C with 5% CO_2_ for 24 hours. Viability of HeLa cells was assessed with CellTiter 96 AQ_ueous_ One Solution Cell Proliferation Assay according to manufacturer’s protocols. The combination of 0.35 µl transfection reagent with 25 nM siRNA resulted in the highest level of transfection (as indicated by decreased viability) with the least amount of transfection-induced cell death (as monitored by viability in control treated cells); these conditions were used in library screening and for subsequent experiments (data not shown).

### siRNA Screen Design

Many factors, such as length of protein knockdown, parasite inoculum, and infection length, were considered in the screen design. Our goal was to specifically identify host factors involved in the middle and end of the infection process, as therapeutics against this portion of the parasite life cycle may be more effective. To this end, we infected HeLa cells with *T. gondii* 24 hours after silencing, when protein depletion was expected to be beginning but not complete for most proteins. Proteins necessary for initial steps of infection, attachment, and invasion would likely be present and would allow infection to occur. The hypothesis was that by the time parasites had initiated infection, protein knockdown would be significant advanced, so that host proteins necessary for middle to later stages of parasite could be identified when parasite growth was slowed or arrested. We used a parasite inoculum of 2.5×10^4^/well to ensure that a single round of infection resulted in host cell lysis between 48 and 72 hpi (Fig. 1) and to prevent over-infection of the monolayer. Over-infection could result in premature lysis, which would likely occur before sufficient protein knockdown and thereby mask the effects of gene silencing.

### siRNA Library

We used the Dharmacon Human siGENOME SMARTpool siRNA Library maintained by the Small Molecule Screening Facility at the University of Wisconsin-Madison. The library contains siRNA pools, each composed of four siRNAs, targeting approximately 18,200 discreet genes. The content of the siRNA pools was proprietary and not sold individually; however, siRNA pools were available for purchase.

### Large-scale siRNA Transfection and Infection Screen

The siRNA library was screened in a 96 well plate format. For each plate of the library, a 96 well black clear bottom plate was seeded with HeLa (7000 cells/well). The cells were incubated at 37°C with 5% CO_2_ for 24 hours to approximately 75% confluency (∼1.6×10^4^ cells/well). In each well of a separate 96 well plate, 0.35 µl of *Trans*IT-TKO Transfection reagent (Mirius Bio) was mixed with 9 µl Opti-MEM and incubated at room temperature for 20 minutes. siRNAs were added to each well from the library plates using a Hydra DT robot (Thermo Scientific) to a final concentration of 25 nM. Plates were incubated at room temperature for 20 minutes and 44 µl of complete growth medium was added to each well. Media was removed and replaced with the siRNA mixture. Columns one and twelve of each plate were used for controls. Qiagen’s AllStars Hs Cell Death Control was applied to wells A12, B12, and C12 and AllStars Negative Control siRNA was added to wells F1, G1, H1. Each plate also contained a well of siGLO RISC-Free control siRNA, a positive control for transfection provided by the manufacturer. Wells G12 and H12 were left uninfected. The plates were incubated at 37°C with 5% CO_2_ for 24 hours. Cells (∼2.5×10^4^/well) were then infected with RHΔHXGPRT::mCherry 9 (2.5×10^4^ parasites/well) in 50 µl of complete growth medium and incubated at 37°C with 5% CO_2_. Fluorescence of each well was read 1–2 hpi (excitation: 589 nm, emission: 615 nm) and every subsequent 24 hours until 72 hpi. If the negative siRNA wells differed significantly in fluorescence from the no siRNA wells or if the death siRNA wells were not significantly lower than negative or no siRNA wells, the plate was discarded and the samples rescreened.

### Data Analysis for Large-scale Screen

Fluorescence readings taken 1–2 hours after *T. gondii* infection were considered background values for each well. Corrected readings were obtained by subtracting background values from the subsequent readings of the corresponding wells. Relative fluorescence was determined by dividing the corrected readings by the average corrected fluorescence from wells containing HeLa cells transfected with AllStars Negative Control siRNA and infected with *T. gondii*. Wells in which the relative fluorescence was less than or equal to 50% of the AllStars Negative Control siRNA control wells were re-examined in three to four additional runs. A 50% reduction in fluorescence was chosen to allow us to identify gene targets with a significant effect on parasite replication. Smaller changes in fluorescence may not represent significant changes in parasite growth. Furthermore, a 50% cutoff yielded a manageable number of gene targets to examine.

### Toxicity Assays for HeLa Cells

During optimization of the transfection protocol, CellTiter 96 AQ_ueous_ One Solution Cell Proliferation Assay was used to assess viability of the host cells. This colormetric method uses a tetrazolium compound [3-(4,5-dimethylthiazol-2-yl)-5-(3-carboxymethoxyphenyl)-2-(4-sulfophenyl)-2H-tetrazolium, inner salt; MTS] and an electron coupling reagent (phenazine ethosulfate; PES). MTS is reduced by viable cells, forming the colored product formazan, which is detectable at 490 nm. AllStars Negative Control siRNA, which has no homology to human DNA, and AllStars Hs Cell Death Control siRNA, which target genes essential for cell survival, were used as controls.

In subsequent runs, the viability of siRNA-treated HeLa cells was determined using the CytoTox-ONE Homogeneous Membrane Integrity Assay (Promega). This assay measures release of lactate dehydrogenase (LDH) as an indicator of cytoxocity. siRNA transfection was performed as stated above using AllStars Negative Control siRNA, AllStars Hs Cell Death Control siRNA, or gene-specific siRNA pools. At the indicated times after transfection, 50 µl of media was removed from each well. Control cells were treated with 1 µl of lysis solution and media collected; this represented the maximum LDH release values. Samples were incubated with 50 µl CytoTox-ONE Reagent at room temperature for 10 min; the reaction was stopped by the addition of 25 µl stop solution. The fluorescence of samples (excitation: 560 nm, emission: 590 nm) was measured using SpectraMax Gemini EM fluorometer. Data were graphed as a percentage of total LDH release.

Cell viability was also monitored by trypan blue exclusion. HeLa cells in a 96 well plate were treated with transfection medium alone or transfected with siRNAs as described above. Every 24 hours post transfection (hpt), media was removed and reserved, the monolayer washed with PBS, and 25 µl of trypsin (Gibco) added. The cells were incubated at 37°C until the monolayer detached and the spent media was added back to neutralize the trypsin. The cell suspension was mixed with an equal volume of trypan blue (Cellgro) and counted on a hemocytometer. At least 50 cells were counted per well and each condition was repeated in quadruplicate.

### Western Blotting

HeLa cells were plated at a density of 1×10^5^ cells per well in a 24 well plate. For siRNA transfection, 50 ul OptiMEM was incubated with 2 µl *Trans*IT-TKO for 20 min at room temperature. siRNAs were added to a final concentration of 25 nM, incubated for 20 min at room temperature, and added to HeLa cells in 250 µl complete media. Cells were incubated at 37°C and samples were collected every 24 hpt up to 96 hours. Unless noted otherwise, whole cell lysates were examined by Western blotting. With the exception of Wee1, which was diluted in 5% BSA, all primary antibodies were diluted in 5% milk and incubated on the membrane overnight at 4°C. Primary antibodies used were as follows: rabbit anti-Wee1, 1∶1000 (Cell Signaling Technology); mouse anti-TAP, 1∶500 (NXF1, Santa Cruz Biotechnology); mouse anti-Tubb4q, 1∶1000 (Abnova); mouse anti-eIF4A3, 1∶5000 (DDX48, Millipore clone 3FI); goat anti-DDX19, 1∶500 (Abgent); rabbit anti-ZO2, 1∶200 (TJP2, Santa Cruz Biotechnology); rabbit anti-Ran-GTP, 1∶5000 (Upstate). After washes, the membranes were incubated with the appropriate HRP-conjugated secondary antibody (1∶5000–1∶10,000) and developed using ECL+ chemiluminescence (GE Healthcare).

### Nuclear Fractionation

HeLa cells in a 24 well plate were transfected with 25 nM of siRNA to DDX19 or NXF1 as described above and incubated at 37°C. Samples were collected every 24 hpt up to 96 hours. The cells were pelleted and resuspended in 200 µl Buffer A, pH 7.9 (10 mM Hepes, 1.5 mM MgCl_2_, 10 mM KCl, 0.5 mM DTT, 0.05% NP40, plus protease inhibitors [cOmplete Mini EDTA-free tablet, Roche]) and incubated for 10 min on ice. The lysate was centrifuged at 3000 RPM for 10 min at 4°C. The supernatant containing the cytoplasmic fraction was removed and the pellet was resuspended in 187 µl Buffer B, pH 7.9 (5 mM Hepes, 1.5 mM MgCl_2_, 0.2 mM EDTA, 0.5 mM DTT, 26% glycerol) plus 13 µl NaCl (4.6 M). The pellet was homogenized by passing the solution through a 1 ml syringe fitted with a 27 gauge needle approximately twenty times. The solution was incubated on ice for 30 min and centrifuged at 13,000 RPM for 20 min at 4°C (www.abcam.com/technical). The supernatant containing the nuclear fraction was collected.

### Statistical Analysis

Student’s t-test was employed to examine significance between samples using either Microsoft Office Excel or Prism. Significance is defined as p<0.05 and is indicated where appropriate by an asterisk (*).

## Results

### Identification of Human Genes Necessary for *T. gondii* Infection using siRNA

**Figure 2:**
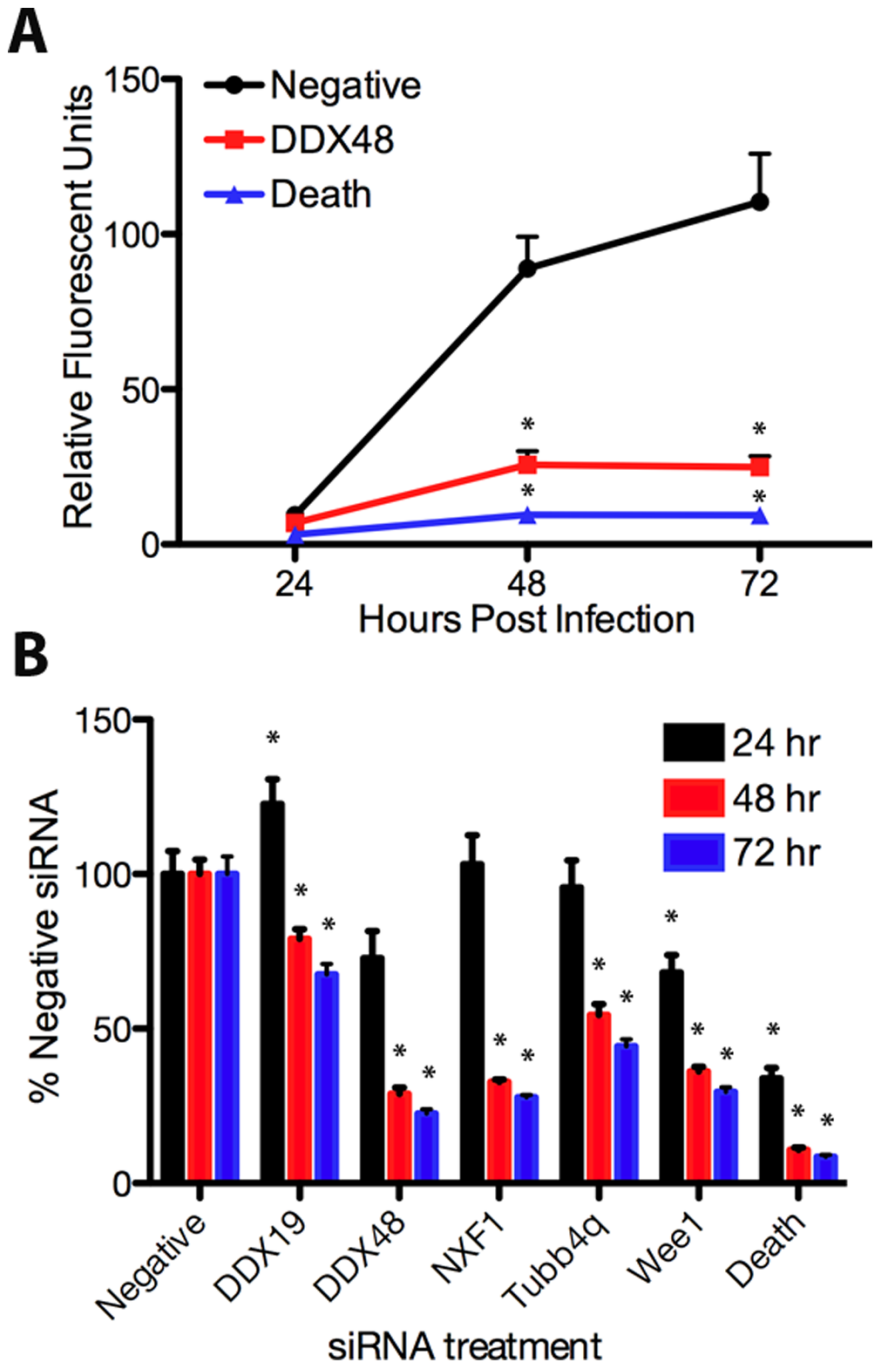
siRNA against human genes inhibits *T. gondii* growth. HeLa cells were transfected with siRNA pools to the indicated gene for 24 hours, then infected with 2.5×10^4^ mCherry-expressing parasites. Fluorescent measurements were taken at 24, 48, and 72 hours post infection. (A) Replication of *T. gondii* as measured by fluorescence in HeLa cells transfected with DDX48 siRNA (DDX48, squares), AllStars Negative Control siRNA (negative, circles), or AllStars Hs Cell Death Control siRNA (death, triangles). (B) Replication of *T. gondii* graphed as the percent growth of wells treated with AllStars Negative Control siRNA (% negative siRNA) at 24 (black), 48 (red), and 72 (blue) hours post infection. Asterisks indicate a significant (p<0.05) difference from negative siRNA at the corresponding timepoint.

In order to determine host genes crucial for *T. gondii* tachyzoites to attach, invade, and replicate in human cells, a genome-wide siRNA screen was performed using fluorescence as an indication of *T. gondii* growth (Table S1). The screen was done in a 96 well plate format to allow for high-throughput processing with one gene silenced per well. siRNAs were incubated with the host cells for 24 hours prior to infection with mCherry-expressing *T. gondii*. Fluorescence measurements were made every 24 hpi, with the largest differences seen at 72 hpi (example in Fig. 2A). Knockdown of a subset of host genes appears to increase *T. gondii* replication (Table S1); however, we chose to focus on genes that decreased growth. Forty-three siRNAs caused at least a 50% reduction in fluorescence at 72 hpi compared to controls in the initial screen (Table 1). These 43 siRNAs were examined in three additional runs using the same procedure as the primary screen. Of the 43 candidates retested, 19 targets showed a 50% decrease in growth in at least three of the four tests (Table 1, bold).

**Table 1:** Host proteins involved in parasite replication.

Gene Ontology	Gene Name	Abbr	Screen	Test 1	Test 2	Test 3
**Cell Cycle**	**WEE1 homolog (** ***S. pombe*** **)**	**Wee1**	**0.494**	**0.146**	**0.026**	**0.421**
**Cell Cycle**	**SON DNA binding protein**	**SON3**	**0.276**	**0.456**	**0.395**	**1.178**
**Cell Cycle**	**RNA binding motif protein 22**	**RBM22**	**0.433**	**0.400**	**0.431**	**0.677**
Cell Cycle	membrane component; chromosome 11;surface marker 1	GPIAP1	0.464	0.425	0.836	0.776
**Cell Cycle**	**kinetochore associated 2**	**HEC1**	**0.297**	**0.328**	**0.700**	**0.424**
**Cytoskeleton**	**tight junction protein 2 (zona occludens 2)**	**TJP2**	**0.447**	**0.461**	**0.168**	**0.726**
Cytoskeleton	nudE nuclear distribution gene E homolog 1(A. nidulans)	NUDE	0.222	1.248	0.206	0.849
Cytoskeleton	tubulin; alpha; ubiquitous	TUBA1B	0.381	0.335	0.663	0.791
**Cytoskeleton**	**tubulin; beta polypeptide 4; member Q**	**TUBB4Q**	**0.213**	**0.192**	**0.523**	**0.645**
Cytoskeleton	ras homolog gene family; member C	RHOC	0.489	0.649	1.171	0.332
Cytoskeleton	actin binding LIM protein family; member 2	ABLIM2	0.422	0.755	0.273	1.375
G-protein coupled receptor	trace amine associated receptor 2	TAAR2	0.270	0.503	0.905	1.056
G-protein coupled receptor	cannabinoid receptor 2 (macrophage)	CNR2	0.429	0.471	0.786	1.366
G-protein coupled receptor	opsin 3 (encephalopsin; panopsin)	OPN3	0.483	0.456	0.916	1.210
G-protein coupled receptor	SH3 and multiple ankyrin repeat domains 3	PSAP-2	0.328	1.272	0.309	0.766
**Proteasome**	**proteasome (prosome; macropain)** **26S subunit; ATPase; 5**	**S5**	**0.457**	**0.561**	**0.463**	**1.134**
**Proteasome**	**proteasome (prosome; macropain)** **26S subunit; non-ATPase; 3**	**S3**	**0.155**	**0.293**	**0.30**	**0.668**
**Proteasome**	**proteasome (prosome; macropain)** **26S subunit; non-ATPase; 1**	**S1**	**0.261**	**0.421**	**0.965**	**0.533**
**Translation**	**DEAD (Asp-Glu-Ala-As) box** **polypeptide 19** **isoform 3**	**DDX19**	**0.314**	**0.677**	**0.456**	**0.546**
**Translation**	**eukaryotic translation initiation factor 4A;**	**eIF4A3**	**0.492**	**0.296**	**0.542**	**1.107**
Translation	ribosomal protein; large; P0	P0	0.167	1.005	0.463	0.709
**Translation**	**ribosomal protein S12**	**RPS12**	**0.212**	**0.265**	**0.338**	**0.500**
Translation	ribosomal protein S10	RPS10	0.273	0.974	0.260	0.761
Translation	Homo sapiens RNA-binding protein withmultiple splicing 2 (RBPMS2); mRNA	RBPMS2	0.200	1.091	0.436	0.931
**Translation**	**nuclear RNA export factor 1**	**NXF1**	**0.258**	**0.155**	**0.86**	**0.262**
Translation	PIH1 domain containing 2	PIHID2	0.406	0.743	2.620	1.011
Transcription	KIAA1194	CCR4	0.414	0.389	1.077	1.134
Transcription	polymerase (RNA) II (DNA directed)polypeptide F	RPB6	0.184	0.174	0.682	0.642
**Transcription**	**homeo box A9**	**HOX1**	**0.475**	**0.441**	**0.546**	**1.082**
Transcription	zinc finger protein 562	ZNF562	0.297	0.994	1.119	0.441
Transcription	integrator complex subunit 12	INTS12	0.190	0.891	1.183	0.328
Ion channels/Receptors	coagulation factor II (thrombin) receptor-like 3	PAR4	0.377	0.348	0.640	1.087
Ion channels/Receptors	potassium channel; subfamily T; member 2	KCNT2	0.209	0.444	0.869	1.230
**Ion channels/Receptors**	**glutamate receptor; ionotropic;** **N-methyl D-aspartate 1**	**GRIN1**	**0.459**	**0.404**	**1.067**	**0.451**
**Ion channels/Receptors**	**CKLF-like MARVEL transmembrane** **domain containing 5**	**CKLFSF5**	**0.397**	**0.386**	**0.510**	**0.560**
Ion channels/Receptors	calcium channel; voltage-dependent;gamma subunit 4	CACNG4	0.409	0.450	0.821	1.223
Ion channels/Receptors	neural precursor cell expressed;developmentally down-regulated 4-like	RSP5	0.490	0.413	1.310	0.912
**Ion channels/Receptors**	**polycystic kidney disease 1** **(autosomal dominant)**	**PKD1**	**0.160**	**0.398**	**0.318**	**0.628**
**Growth Factor**	**bone morphogenetic protein 4**	**BMP4**	**0.389**	**0.403**	**0.543**	**0.787**
Lipid Metabolism	START domain containing 3	STARD3	0.464	0.626	0.702	0.352
**Protein Transport**	**archain 1**	**ARCN1**	**0.192**	**0.283**	**0.327**	**0.368**
Unknown; ER membrane associated	transmembrane andtetratricopeptide repeatcontaining 2	TMTC2	0.423	0.794	0.310	1.000
Unknown	hypothetical protein MGC40179	MGC40179	0.366	0.420	0.692	1.201

This table lists the human gene targets that, when silenced, reduce parasite replication by at least 50% in the initial library screen. Common abbreviations or alternative names as well as the fold change in parasite growth in each test are provided. Genes in bold had at least three runs with approximately 50% or more reduction in parasite growth.

We chose five of these host genes to investigate further: the cell cycle protein Wee1, the helicases DDX19 and DDX48, nuclear export factor 1 (NXF1), and a member of the tubulin family of structural proteins, tubulin-4q (Tubb4q). Wee1 was chosen for its role in cell cycle control (Pendergast 1996), as *T. gondii* is known to preferentially infect cells in S phase as well as to promote cycling of quiescent cells to S phase [16], [17]. The fact that the screen yielded three members of the RNA export pathway –DDX19, DDX48 and NXF1– made those members potentially interesting. *T. gondii* actively disrupts and rearranges the microtubule network around the parasitic vacuole [18], suggesting that disruption of a structural protein like Tubb4q may limit parasite replication.

### Confirmation of Screen Findings

We obtained the siRNA pools of the chosen targets from Dharmacon and confirmed their effects in a 96 well plate format, as used for the original screen (Fig. 2B). In each case, the phenotype became more pronounced over time, with the most drastic reduction in parasite growth occurring between 24 and 48 hpi. However, the kinetics and the degree of parasite reduction differed among siRNA treatments. Growth in cells treated with DDX19, NXF1, and Tubb4q siRNAs was relatively unaffected at 24 hpi, while treatment with siRNAs targeting DDX48 and Wee1 resulted in an early reduction in parasite growth. By 72 hpi, cells treated with siRNAs to DDX48, NXF1, and Wee1 supported parasite growth of only 20–30% of the negative control. Cells treated with DDX19 or Tubb4q siRNAs demonstrated a more intermediate phenotype with parasite growth reduced by 32% and 65%, respectively. HeLa cells were also transformed with AllStars Cell Death Control siRNA as a positive control. HeLa cells transfected with AllStars Cell Death Control siRNAs show an approximately 70% drop in fluorescence at 24 hpi compared to untreated controls. Fluorescence levels dropped through 72 hpi when they reached 8.5% of controls. Because *T. gondii* is an obligate intracellular pathogen, death of the host cell was expected to reduce growth.

### Depletion of Targeted Protein

**Figure 3:**
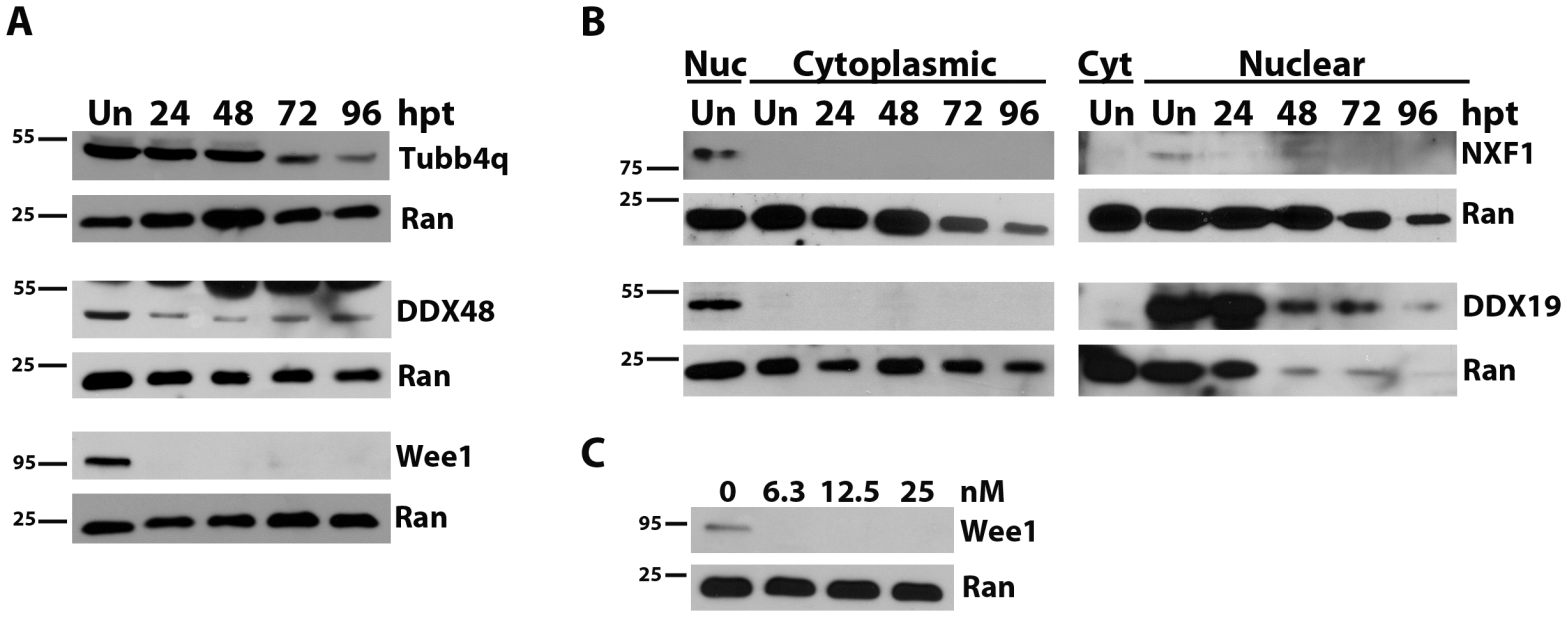
Transfection with siRNA reduces targeted protein over time. HeLa cells were untransfected (Un) or transfected with 25 nM siRNA to the indicated gene. Protein samples were subjected to Western immunoblotting and membranes probed for the protein of interest. Membranes were probed for Ran-GTP (Ran) as a loading control. Size markers in kDa are indicated to the left of each blot. At 24, 48, 72 and 96 hours post transfection (hpt), total protein was collected (A) or cells were fractionated into cytoplasmic (cyt) and nuclear (nuc) fractions (B). Panel C, HeLa cells were transfected with 0–25 nM siRNA to Wee1 and total protein collected at 24 hpt.

To examine whether the decrease in parasite replication corresponded with depletion of the targeted protein, cellular protein was collected at 24, 48, 72 and 96 hpt, and levels of the protein of interest was examined by Western immunoblotting. RNA silencing had an early and drastic effect on Wee1 expression. The protein was undetectable in the total cell lysates by 24 hpt and through 96 hpi (Fig. 3A). Overexposure of the blot demonstrated very low levels of protein at these timepoints (data not shown). In contrast, only a slight decrease in protein levels was observed in cells transfected with siRNAs to Tubb4q at 48 hpt. Protein levels did not appreciably decrease until 72 hours (Fig. 3A). DDX48 protein levels were decreased by 24 hpt and remained at this decreased level through 96 hours (Fig. 3A). All blots were probed for Ran GTPase, which cycles between the nucleus and the cytoplasm and is easily detected in both fractions [19], to verify equal loading.

NXF1 and DDX19 could not be detected in total cell lysates (data not shown). These proteins shuttle between the nucleus and the cytoplasm, but are primarily found in the nucleus. siRNA treated cells were therefore fractionated and the nuclear proteins examined by Western blotting. Cells treated with siRNAs to NXF1 showed early and sustained silencing. NXF1 was identifiable in untreated nuclear but not cytoplasmic fractions (Fig. 3B). Low levels were detectable in nuclear fractions of siRNA-treated cells at 24 hpt but barely detectable at subsequent timepoints. A slight knockdown in DDX19 protein levels was apparent by 24 hours and almost complete protein knockdown was achieved by 72 hours (Fig. 3B). Protein levels decreased through the siRNA time course; however, levels of the loading control protein Ran GTPase was also low at later timepoints. Although NXF1 shuttles between the cytoplasm and the nucleus [20] and DDX19 associates with the cytoplasmic side of the nuclear envelope [21], neither of these proteins were detected in cytoplasmic fractions (Fig. 3B).

### siRNA Treatment Increases HeLa Cell Death

**Figure 4:**
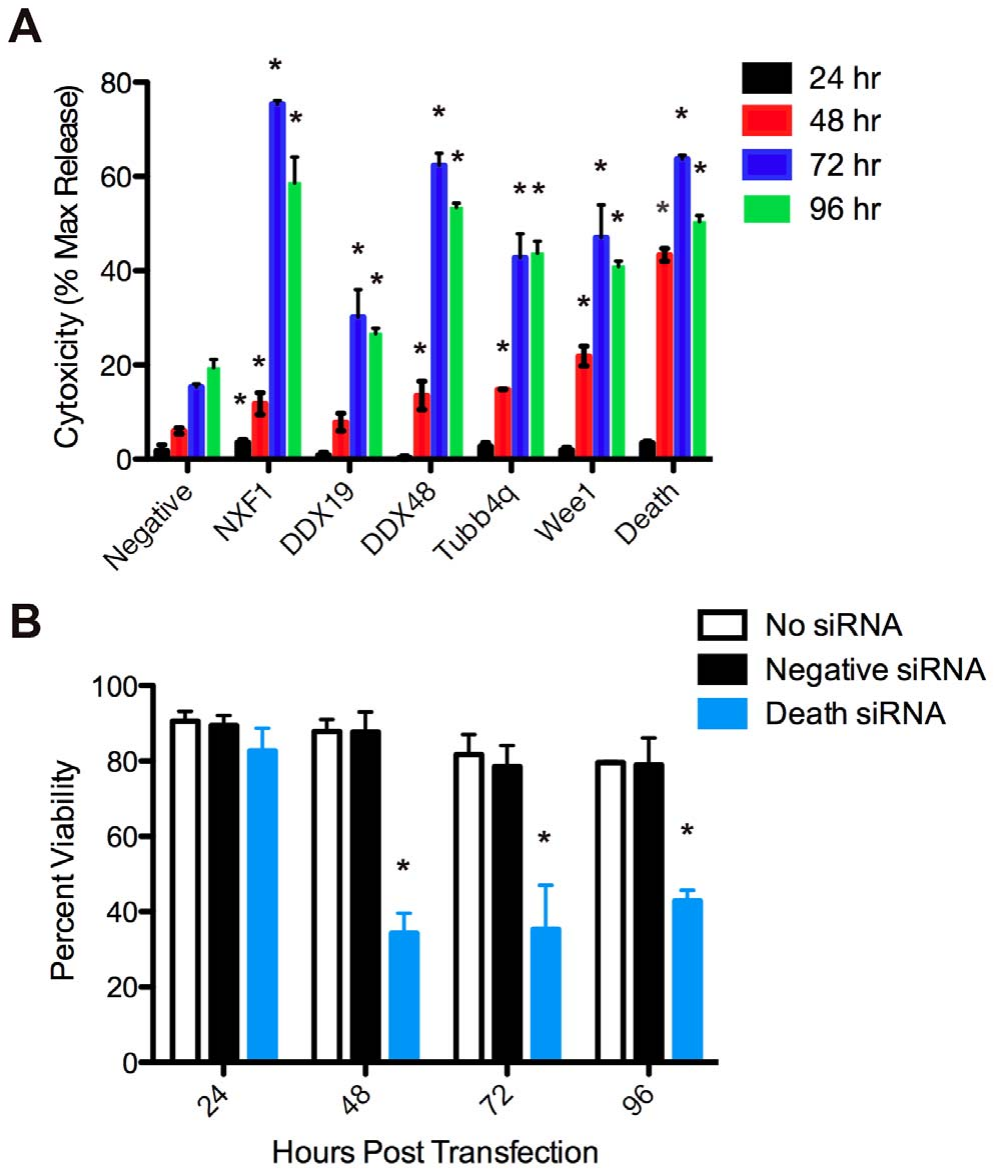
siRNAs, not transfection alone, induces host cell toxicity over time. (A) At 24, 48, 72 and 96 hours post transfection, medium from HeLa cells transfected with 25 nM siRNA pools was mixed with an equal volume CytoToxONE assay reagent. Fluorescence of samples was measured and data plotted as a percentage of the maximum LDH release obtained from lysed cells (% max release). (B) HeLa cells were treated with transfection medium alone (white) or transfected with AllStars Negative Control (black) or AllStars Hs Cell Death Control (blue) siRNAs. At the indicated times, cells were trypsinized and the number of viable cells determined by trypan blue exclusion. Asterisks indicate a significant (p<0.05) increase from negative siRNA wells (A) or decrease from no siRNA control wells (B) at the corresponding timepoint.

We next examined whether transfecting siRNAs had any effect on viability of the host cell in order to evaluate cell death as a mechanism for decreased parasite replication. HeLa cells were transfected with siRNAs and supernatants were examined every 24 hpt for LDH, an indicator of cell death. Cells treated with a AllStars Negative Control siRNA showed little cytotoxicity (1.8–15%), while cells transfected with AllStars Cell Death Control siRNA demonstrated 50–60% death from 48–96 hpt (Fig. 4A). The effect on cells transfected with siRNA varied depending on the siRNA target, but cytotoxicity generally increased over time. Less than 5% cytotoxicity was observed in any sample at 24 hpt. By 48 hours, cells treated with Wee1 siRNAs demonstrated cytotoxicity levels greater than 20%, while those treated with NXF1, DDX19, DDX48, and Tubb4q remained less than 15%. Cytotoxicity levels peaked at 72 hpt. siRNA silencing of DDX48 and NXF1 led to cytotoxicity over 60%, similar to that caused by cell death siRNAs. Treatment with Tubb4q or Wee1 siRNAs caused less drastic cytotoxicity, resulting in ∼45% death, and treatment with DDX19 caused the least cytotoxicity of the experimental siRNAs (30%). A similar pattern was observed at 96 hpt, although the values were slightly lower than at 72 hpt. This decrease at 96 hours is likely due to the instability of LDH, which has a half-life of 9 hours, and suggests that the peak cytotoxicity occurs at or around 72 hpt. This 72 hpt time point corresponds to 48 hpi in the parasite growth assay and perhaps contributes to the pronounced effects on parasite replication observed at 72 hpi (96 hpt).

To verify that decreases in cell viability were not due to the transfection process itself and to estimate the percentage of cells transfected, HeLa cells were transfected with either AllStars Negative Control siRNA or AllStars Cell Death Control siRNAs or treated with transfection medium lacking siRNAs. Cell integrity was measured by trypan blue exclusion every 24 hours through 96 hpt. No difference in cell viability was observed between cells treated with transfection medium alone and cells transfected with AllStars Negative Control siRNA (Fig. 4B), demonstrating that the transfection process was not responsible for cell lethality. Approximately 35% viability was observed in cells transfected with AllStars Cell Death Control siRNA from 48 hpt to 96 hpt. These data suggest that the transfection efficiency in these studies is at least 65%, as death likely occurs in most cells transformed with death siRNAs.

## Discussion

Obligate intracellular parasites develop intimate interactions with their host in order to establish an infection, replicate, and persist. In an effort to identify host proteins involved in initiating and maintaining a *T. gondii* infection, we employed an RNAi approach for a large-scale, high-throughput screen. While a large number of host knockdowns appear to increase *T. gondii* replication (Table S1), we chose to focus on genes whose knockdown decreased growth. Nineteen genes involved in a variety of cell processes were found to decrease parasite growth following transfection with homologous siRNAs. Our low hit percentage (0.09%) for genes that decreased *T. gondii* replication is likely because the time period between siRNA transfection and *T. gondii* infection in our study, 24 hours, is relatively short and may not allow for sufficient knockdown of all proteins. The rate of protein depletion is dependent on the abundance and stability of a given protein. Proteins that turn over more quickly will be silenced faster than proteins with a long half-life. For example, Wee1 protein levels and activity increase during S and G2 phases; however, to progress to M phase, Wee1 must be degraded [22], [23]. In our study, Wee1 was completely depleted by 24 hours (Fig. 3A); this phenotype was evident even when lower concentrations of siRNA against Wee1 were used (Fig. 3C). Rapid protein depletion is a particularly important consideration when examining early stages of the lifecycle, such as binding or entry, in which the host protein may not be required once the infection is established. Many studies try to avoid this issue by separating transfection and infection by 48–72 hours in order to allow proteins to be knocked down efficiently. By selecting only 24 hours between siRNA transfection and *T. gondii* infection, we likely missed proteins important for attachment and invasion. It is possible that additional proteins, especially those involved in early stages of infection, could be recovered by extending the time between transfection and infection.

**Figure 5:**
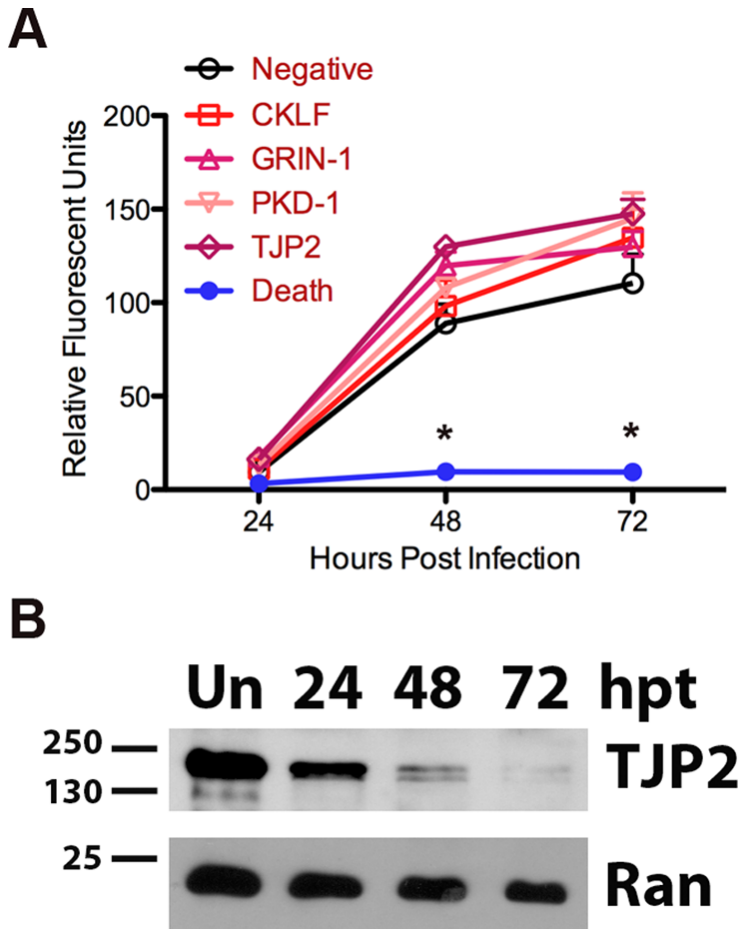
siRNA against TJP2 reduces protein but does not inhibit *T. gondii* growth. (A) HeLa cells were transfected with 25 nM siRNA to TJP2 (diamonds), CKLF (square), GRIN-1 (triangle), PKD-1 (inverted triangle), AllStars Hs Cell Death Control siRNA (death, closed circles), or AllStars Negative Control siRNA (negative, open circles) for 24 hours, then infected with 2.5×10^4^ mCherry-expressing parasites. Replication of *T. gondii* over time was measured by fluorescence. Asterisks indicate a significant (p<0.05) decrease from negative siRNA wells at the corresponding timepoint. (B) HeLa cells were untransfected (Un) or transfected with 25 nM TJP2 siRNA. At 24, 48, and 72 hours post transfection (hpt), total cell protein was collected and examined by Western immunoblotting. Membrane was probed for Ran-GTP (Ran) as a loading control. Size markers in kDa are indicated on the left.

It is important to note that the effects of a subset of genes identified in the library screen did not reproduce when siRNA pools were purchased. siRNA pools to tight junction protein 2 (TJP2), glutamate receptor; ionotropic; N-methyl D-aspartate 1 (GRIN-1), polycystic kidney disease-1 (PKD-1), and CKLF-like MARVEL transmembrane domain containing 5 (CKLF) were purchased from Dharmacon and transfected into HeLa cells under conditions identical to those used in the library screen. Treatment with these siRNAs failed to decrease *T. gondii* replication (Fig. 5A). Attempts to monitor protein knockdown for CKLF, PKD-1 and GRIN-1 by Western immunoblotting failed; although we used commercial antibodies, the specific protein of interest could not be identified in the samples. siRNAs against TJP2 decreased protein levels, modestly at 24 hpt and significantly by 48 and 72 hpt (Fig. 5B), indicating that the siRNAs were functional even though no effect on parasite replication was observed. siRNA pools have the advantage of producing a more pronounced phenotype and a higher hit frequency than do individual duplex siRNAs [24]. The Dharmacon SMARTpool siRNA library format contains four siRNA duplexes per target gene to minimize off-target effects. Each duplex is at a concentration below that required for efficient knockdown; only in combination can effective knockdown be achieved. After we screened the library, Dharmacon updated the commercially available human siRNA pools to further minimize off-target effects. In this subset of genes, it seems likely that off-target effects were responsible for the observed phenotype during the initial screen and that the updated pools failed to replicate the growth inhibition originally observed.

Of the 19 genes identified, three play an important role in mRNA export. DDX48 contributes to the assembly of export ready mRNA:protein complexes [25]. This complex includes NXF1, which binds polyadenylated RNA transcripts and targets the mRNA:protein complex to the nuclear pore complex [26], [27]. At the nuclear pore complex, NXF1 transfers the RNA to DDX19 [26]; the energy provided in ATP hydrolysis by DDX19 translocates the RNA to the cytoplasm, where it can be translated [28], [29]. It is possible that hits for these three genes merely reflect disruption of RNA export in the host cell. Inhibition at any one of these steps may restrict protein synthesis to such a degree that overall host cell health suffers, leading to cell death and an inability to support parasite growth. Our cell viability data lends support to this possibility, as a significant decrease in cell viability was observed by 48 hours after treatment with each set of siRNAs.

As an obligate intracellular parasite, *T. gondii* requires viable host cells to survive. *T. gondii* actively invades host cells in a well described process that uses both host cell and parasitic proteins (reviewed in [30]). However, once the *T. gondii* has invaded the host cell, it is surrounded by the parasitophorous vacuole membrane, which limits communication between the host cell and the parasitophorous vacuole. *T. gondii* proteins, such as ROP16, PP2C-hn, and possibly GRA10, have been localized inside the host cell nucleus [31], [32], [33] although host proteins have not been identified within the parasitophorous vacuole. It is possible at this stage that the parasite is primarily self-sufficient, like a virus, relying on the host for nutrients more than on cellular processes and modulating the host environment to prevent detection. Perhaps only the drastic global changes associated with cell death are sufficient to limit *T. gondii* replication once the parasite has established an infection. This phenomenon may explain why the hits we identified uniformly induced cell death.

It is important to note that the RH strain of *T. gondii* used in this study belongs to the type I lineage. Type I parasites are more virulent than type II or type III strains and differ in important infection characteristics such as growth rate and ability to establish chronic infections in hosts (reviewed in [34]). Differences in host gene regulation have been also described for the different lineages [35] and it is not unlikely that a screen similar to the one described here performed with a less virulent strain would yield different results.

### Conclusions

Gene silencing by siRNA transfection has been used extensively to identify cellular proteins important in host/cell interactions. The majority of published studies have focused on viral and bacterial pathogens. Few studies have used siRNA to dissect host involvement during parasite infection and the ones that have report a lower number of hits compared to the viral and bacterial studies. This low hit percentage may reflect the degree to which the pathogen is dependent on the host. For example, viruses exclusively require host cell machinery to replicate, while parasites such as *T. gondii* mostly mine resources from the host cell environment. It logically follows that more host cell proteins play a role in achieving a productive viral infection than a parasitic infection. In light of this, it may be most pragmatic for future studies to focus on identifying parasite proteins involved in the initial steps of attachment and invasion or modulating the host environment rather than on identification of host cell proteins required for parasite maintenance. Extending the silencing time of host cell genes to 48 hours prior to *T. gondii* infection may also reveal additional host genes involved early in the infection process.

## Supporting Information

Table S1 **Complete results of human siRNA library screen.** Genes in the human siRNA library are grouped by target similarity. Provided is the gene name, abbreviation, accession number and fold change in parasite growth at 72 hours post-infection.
(XLSX)
